# Strong *In Vivo* Inhibition of HIV-1 Replication by Nullbasic, a Tat Mutant

**DOI:** 10.1128/mBio.01769-19

**Published:** 2019-08-27

**Authors:** Hongping Jin, Yifan Sun, Dongsheng Li, Min-Hsuan Lin, Mary Lor, Lina Rustanti, David Harrich

**Affiliations:** a Department of Cell and Molecular Biology, QIMR Berghofer Medical Research Institute, Herston, QLD, Australia; Medical School, University of Athens

**Keywords:** Nullbasic, animal models, antiviral agents, gene transfer, human immunodeficiency virus

## Abstract

Nullbasic is a mutant form of the HIV-1 transcriptional activator protein (Tat) that strongly inhibits HIV-1 transcription and replication in lymphocytes *in vitro*. To investigate Nullbasic inhibition *in vivo*, we employed an NSG mouse model where animals were engrafted with primary human CD4^+^ cells expressing a Nullbasic-ZsGreen1 (NB-ZSG) fusion protein or ZSG. NB-ZSG and ZSG were delivered by using a retroviral vector where CD4^+^ cells were transduced either prior to (preinfection) or following (postinfection) HIV-1 infection. The transduced cells were analyzed *in vitro* up to 10 days postinfection (dpi) and *in vivo* up to 39 dpi. Compared to ZSG, NB-ZSG strongly inhibited HIV-1 replication both *in vitro* and *in vivo* using preinfection treatment. *In vitro*, HIV-1 mRNA levels in cells were reduced by up to 60-fold. *In vivo*, HIV-1 RNA was undetectable in plasma samples during the course of the experiment, and HIV-1 mRNA levels in resident CD4^+^ cells in organ tissue were reduced up to 2,800-fold. Postinfection treatment of HIV-1-infected cells with NB-ZSG attenuated HIV-1 infection for up to 14 days. *In vitro*, a 25-fold reduction of viral mRNA in cells was observed but diminished to a <2-fold reduction by 10 dpi. *In vivo*, HIV-1 RNA was undetectable in plasma of NB-ZSG mice at 14 dpi but afterwards was not significantly different between NB-ZSG mice and control mice. However, we observed higher levels of CD4^+^ cells in NB-ZSG mice than in control mice, suggesting that NB-ZSG imparted a survival advantage to HIV-1-infected animals.

## Abstract (precis)

HIV-1 infection is effectively controlled by antiviral therapy that inhibits virus replication and reduces viral loads below detectable levels in patients. However, therapy interruption leads to viral rebound due to latently infected cells, which serve as a source of continued viral infection. Interest in strategies leading to a functional cure for HIV-1 infection by long-term or permanent viral suppression is growing. Here, we show that a mutant form of the HIV-1 Tat protein, referred to as Nullbasic, inhibits HIV-1 transcription in infected CD4^+^ cells *in vivo*. Analysis shows that stable expression of Nullbasic in CD4^+^ cells could lead to durable anti-HIV-1 activity. Nullbasic, as a gene therapy candidate, could be a part of a functional-cure strategy to suppress HIV-1 transcription and replication.

## INTRODUCTION

Gene transfer approaches to block human immunodeficiency virus type 1 (HIV-1) infection or inhibit viral replication in cells have tested an array of agents, such as HIV-1-specific antibody mimetics, ribozymes, small interfering RNA (siRNA), short hairpin RNA (shRNA), gp41-based peptides C46 and C34, zinc finger nucleases to CCR5, CRISPR-Cas9 targeting HIV-1 and cellular genes, and various dominant negative mutant forms of HIV-1 Rev and Tat (1–5). Early HIV-1 gene transfer clinical trials used retrovirus-based vectors to deliver a mutant HIV-1 *rev* called M10, a dominant negative inhibitor of wild-type *rev* (6, 7). M10 interferes with the transport of unspliced and singly spliced viral mRNA by Rev from the nucleus to the cytoplasm. In clinical trials investigating HIV-1-positive (HIV-1^+^) subjects, M10-treated T lymphocytes survived preferentially *in vivo*, suggesting that M10 protected cells in infected individuals (8–10). More recently, clinical trials have advanced a zinc finger nuclease that targets CCR5 and renders treated CD4^+^ T cells resistant to HIV-1 infection (4). Other viral entry inhibitors have fused a portion of the gp41 heptad repeat 2 region, a 34-amino-acid peptide, to the amino terminus of CCR5 or CXCR4 and were shown to protect human primary T cells (11) or, in combination with an siRNA targeted to CCR5, were protective in pigtailed macaques against simian-human immunodeficiency virus (SHIV) challenge (12). Finally, other recent approaches have used CRISPR-Cas9 editing of the cellular gene encoding the CXCR5 HIV-1 coreceptor and chimeric antigen receptor (CAR) T cell therapy for HIV-1 using engineered cells that can resist HIV-1 infection and train the immune system to eliminate HIV-1-infected cells (13–15). Clearly, application of therapeutic gene transfer approaches to HIV-1 therapy is progressing.

Our group has investigated a Tat transdominant negative mutant referred to as Nullbasic. Nullbasic is a 101-amino-acid Tat mutant in which the basic domain region residues 49 to 57 are replaced with the amino acids GGGGGAGGG (16). Unlike most transgene inhibitors of HIV-1 that have a single mechanism of action, Nullbasic disrupts three independent steps of virus replication: viral transcription by RNA polymerase II, Rev-mediated transport of viral mRNA, and reverse transcription (RT) of viral genomic RNA into double-stranded DNA (16, 17). Nullbasic inhibits HIV-1 transcription by competing with Tat for binding to the positive transcription elongation factor P-TEFb (17), and further analysis revealed that transcriptional “shutdown” by Nullbasic is associated with chromatin modification within the long terminal repeat (LTR) promoter characteristic of inactive heterochromatin (18). Nullbasic also inhibits Rev function through a direct interaction with DDX1 and results in a redistribution of Rev localization in cells from the nucleolus and nucleoplasm to the nucleoplasm and cytoplasm (19). *In vitro* experiments indicate that Nullbasic inhibits HIV reverse transcription by binding to the viral reverse transcriptase in the virion to destabilize the viral core structure (20).

After gene transfer and stable expression of Nullbasic in Jurkat cells, HIV-1 replication was completely inhibited (18). Nullbasic also completely blocked HIV-1 reactivation from latency in Jlat 6.3 cells treated with phorbol ester 12-myristate 13-acetate (PMA), suberanilohydroxamic acid (SAHA), and JQ-1 (18). When expressed in human primary CD4^+^ cells with a retroviral vector, Nullbasic had strong antiviral activity against HIV-1 strains of different subtypes *in vitro* (21, 22).

To follow up on these interesting outcomes from studies performed *in vitro*, human CD4^+^ cell protection by Nullbasic was tested in a small-animal model of HIV-1 infection. An immunodeficient mouse strain, NOD.Cg-PrkdcscidIl2rgtm1Wjl/SzJl (NSG) (23, 24), was used in two ways. First, CD4^+^ cells were transduced with Nullbasic, sorted, infected with HIV-1, and then engrafted into mice (preinfection treatment). In a second approach, CD4^+^ cells were infected with HIV-1 first, and Nullbasic was then delivered into the infected cells with a retroviral vector (postinfection treatment), sorted, and engrafted into mice.

Our results showed that preinfection treatment with Nullbasic strongly inhibited HIV-1 replication *in vivo* in the CD4^+^ cell-engrafted animals (CD4^+^ mice), with no detectable HIV-1 RNA in animal blood and a 2- to 3-log reduction in HIV-1 mRNA levels in organs compared to HIV-1-infected CD4^+^ and mock-treated mice, respectively. In animals engrafted with postinfection-treated CD4^+^ cells, Nullbasic delayed HIV-1 replication in blood and decreased HIV-1 replication in the organs. The difference in HIV-1 responses to Nullbasic in the two models is interesting and indicates that Nullbasic is a more effective inhibitor in cells prior to the expression of viral proteins.

## RESULTS

### Preinfection treatment of primary CD4^+^ cells with NB-ZSG strongly inhibits HIV-1 replication *in vitro*.

**FIG 1:**
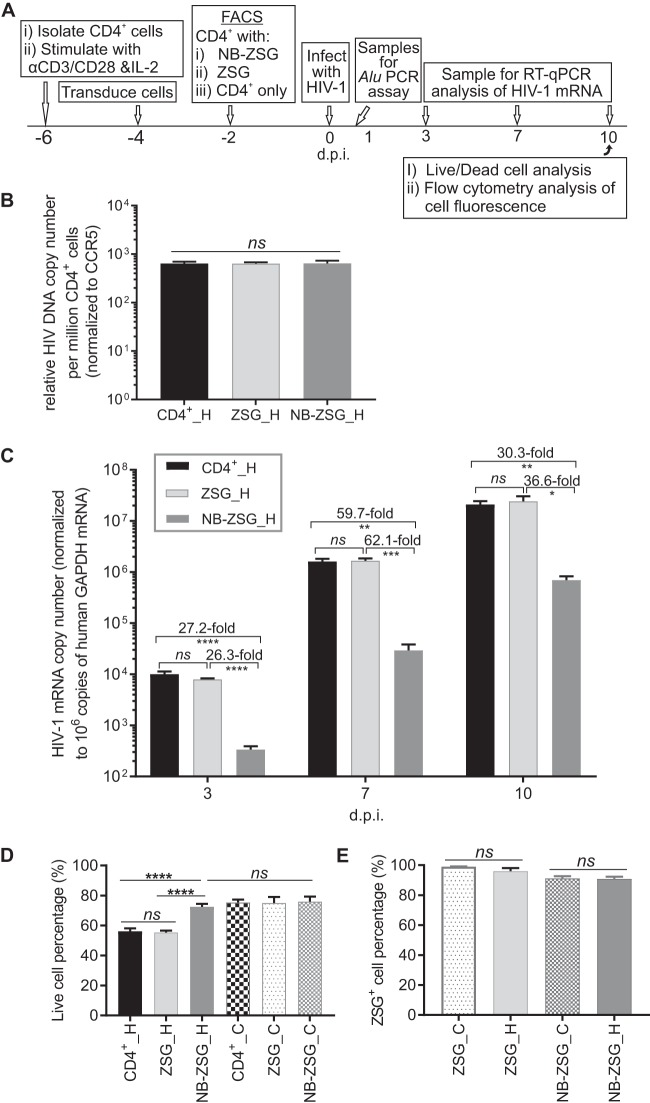
*In vitro* analysis of transduced NB-ZSG and ZSG and nontransduced CD4^+^ cells used to engraft mice. (A) Schematic showing the experimental design to obtain CD4^+^-only cells and transduced CD4^+^ cells expressing NB-ZSG or ZSG by FACS analysis. Samples of the CD4^+^ cells were assayed for HIV-1 infection *in vitro* at 16 h p.i. and at 3, 7, and 10 dpi. (B) Relative level of integrated HIV-1 DNA measured in CD4^+^ cells at 16 h p.i. normalized to CCR5 levels in the DNA sample. (C) HIV-1 mRNA levels in infected cells (designated _H) were measured by RT-qPCR. On the days indicated, total cellular RNA was assayed for the level of HIV-1 mRNA, which was normalized to the level of human GAPDH mRNA in the sample. Duplicate RNA samples were assayed in triplicate from three independent experiments. (D and E) HIV-1-infected (designated _H) and uninfected (designated _C) CD4^+^ cells expressing NB-ZSG or ZSG or CD4^+^-only cells were analyzed by a live/dead cell assay (D) and by flow cytometry (E) at 10 dpi. Samples were assayed in duplicate from three independent assays. The mean values and SD are shown. Statistical significance is indicated (*, *P* < 0.05; **, *P* < 0.01; ***, *P* < 0.001; ****, *P* < 0.0001; *ns*, not statistically different). d.p.i., days postinfection.

We previously showed that NB-ZSG had anti-HIV-1 activity in primary human CD4^+^ cells *in vitro* (21, 22). To confirm and extend those findings, NB-ZSG was delivered into human primary CD4^+^ cells by using a retroviral vector (25), and ZSG was used as a control. The experimental procedure is summarized in Fig. 1A. The NB-ZSG- and ZSG-positive CD4^+^ cell populations were enriched by fluorescence-activated cell sorter (FACS) analysis, and nontransduced cells were also collected, which were used as a control in these experiments. The transduced and nontransduced cells were infected with HIV-1. Next, the HIV-1-infected cells were sampled at 16 h postinfection (p.i.) for *Alu* PCR analysis and at 3, 7, and 10 days postinfection (dpi) to analyze the HIV-1 mRNA levels. The cell samples collected at 10 dpi were assayed for viability and NB-ZSG or ZSG expression.

Genomic DNA samples were extracted from the cells collected at 16 h p.i. and analyzed by using HIV-1 *Alu* PCR assays. As shown in Fig. 1B, the relative levels of the integrated HIV-1 proviral DNA in the infected NB-ZSG-treated CD4^+^ cells (NB-ZSG cells), ZSG-treated CD4^+^ cells (ZSG cells), and CD4^+^ cells were not significantly different.

At 2 dpi, a majority of NB-ZSG, ZSG, and CD4^+^ cells were engrafted into animals, and the remaining cells were grown *in vitro*. Total cellular RNA was extracted from cell samples collected at 3, 7, and 10 dpi, and the levels of viral mRNA, relative to the levels of cellular glyceraldehyde-3-phosphate dehydrogenase (GAPDH) mRNA in the same sample, were measured. As shown in Fig. 1C, CD4^+^ cells expressing NB-ZSG had significantly reduced levels of HIV-1 mRNA at all time points compared to ZSG and nontransduced CD4^+^ cells. Overall, cells expressing NB-ZSG had 26- to 60-fold-reduced levels of viral mRNA compared to cells expressing ZSG or nontransduced CD4^+^ cells. Cells collected at 10 dpi were also analyzed for live-cell populations by using a fluorometric cell assay. As shown in Fig. 1D, about 70% of NB-ZSG cells were alive, which was significantly higher than in HIV-1-infected ZSG cells and nontransduced CD4^+^ cells, where ∼54% cells were alive. There was no statistical difference in live-cell percentages between HIV-1-infected NB-ZSG cells and any of the uninfected CD4^+^ cell groups. At 10 dpi, ZSG fluorescence-positive cell percentages were >89.8% for NB-ZSG cells and >93.7% for ZSG cells (Fig. 1E). NB-ZSG mRNA levels were measured by using RT-quantitative PCR (qPCR) analysis with oligonucleotide primers specific for NB and ZSG sequences. As shown in Fig. S1 in the supplemental material, levels of NB-ZSG mRNA in HIV-1-infected and uninfected cells were similar when measured by using oligonucleotide primers specific for either NB or ZSG sequences. The ZSG mRNA levels in ZSG cells were ∼2.5-fold higher than the NB-ZSG mRNA levels in cells measured by using ZSG oligonucleotide primers. The results here support and extend our previous results showing that NB-ZSG inhibits HIV-1 replication in primary CD4^+^ cells (21, 22).

10.1128/mBio.01769-19.2 FIG S1 NB-ZSG mRNA levels in CD4^+^ cells. Cellular RNA was extracted from HIV-1-infected and uninfected cells and analyzed using RT-qPCR with oligonucleotide primers specific for NB and ZSG sequences. CD4^+^ cells expressing ZSG mRNA and unmodified CD4^+^ cells were included in the assays as controls. The levels of NB-ZSG mRNA shown were all normalized to the level of human GAPDH mRNA in the samples. The results are presented as mean values ± SD. Download FIG S1, EPS file, 0.4 MB. Copyright © 2019 Jin et al. 2019 Jin et al. This content is distributed under the terms of the Creative Commons Attribution 4.0 International license.

### Preinfection treatment of human CD4^+^ cells with NB-ZSG strongly inhibits HIV-1 replication in transplanted mice.

Approximately 4 million HIV-1-infected or uninfected cells were injected into each NSG mouse. Groups of 6 animals were engrafted with NB-ZSG, ZSG, or nontransduced HIV-1-infected CD4^+^ cells, and groups of 4 animals were engrafted with the corresponding cells that were uninfected. Blood samples were collected weekly from each animal for analysis of cell engraftment and viral RNA in plasma. Animals were sacrificed after 31 dpi, and the liver, lung, spleen, and kidney were collected from each animal to measure levels of human CD4^+^ cells in tissue and viral mRNA levels in cells.

**FIG 2:**
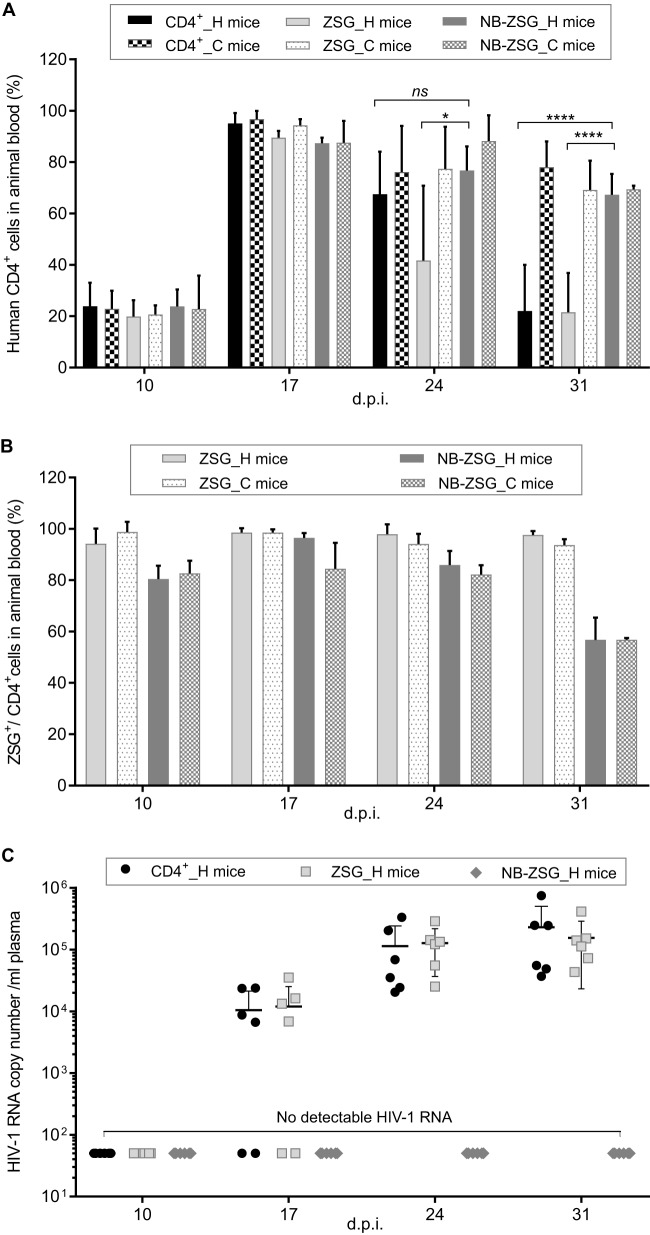
Analysis of blood samples from mice engrafted with CD4^+^ cells expressing NB-ZSG and ZSG and unmodified CD4^+^ cells. Animals were engrafted with 4 million HIV-1-infected (designated _H) (*n* = 6 mice) or uninfected (designated _C) (*n* = 4 mice) human CD4^+^ cells. (A and B) Blood samples were collected weekly from each animal and analyzed for the percentage of human CD4^+^ cells in the samples using an anti-human CD4-APC antibody (A) and ZSG fluorescence in the same samples (B). (C) HIV-1 RNA levels in plasma samples were quantified by RT-qPCR for up to 31 dpi. The mean values and SD are shown. Statistical significance is indicated (*, *P* < 0.05; ****, *P* < 0.0001; *ns*, not statistically different).

The levels of human CD4^+^ cells in animal blood were measured by staining cell samples with an anti-human CD4-allophycocyanin (APC) antibody followed by flow cytometry analysis. Human CD4^+^ cells were detectable in all blood samples at 10 dpi, and peak levels were reached at 17 dpi, when >85% of the cells measured by flow cytometry were human CD4^+^ cells (Fig. 2A). At 24 dpi, CD4^+^ cell percentages in blood samples from HIV-1-infected ZSG and CD4^+^ cell-engrafted mice declined, while the percentage of CD4^+^ cells in HIV-1-infected NB-ZSG mice was significantly higher than that in HIV-1-infected ZSG mice (Fig. 2A). At 31 dpi, the HIV-1-infected NB-ZSG mice had a significantly higher CD4^+^ cell percentage in their blood samples than did the infected ZSG and CD4^+^ mice (Fig. 2A).

The ZSG fluorescence of NB-ZSG and ZSG cells was monitored by flow cytometry. As shown in Fig. 2B, there was no significant difference in the mean percentages of ZSG-positive cells in blood samples from HIV-1-infected compared to uninfected ZSG mice or from HIV-1-infected compared to uninfected NB-ZSG mice. The mean percentage of ZSG-positive cells was >94% in the ZSG mice for all time points. In NB-ZSG mice, the mean percentage of ZSG-positive cells was >80% up to 24 dpi and then decreased to 56% at the last time point. This reason for the decreased NB-ZSG expression at 31 dpi compared to the earlier time point is unknown.

The mouse plasma samples were analyzed for levels of HIV-1 RNA, and the results are shown in Fig. 2C. HIV-1 RNA was not detected by RT-qPCR at the first time point in all animals. At 17 dpi, HIV-1 RNA was measurable in 4 of the 6 mice from the HIV-1-infected ZSG and CD4^+^ groups. In HIV-1 RNA-positive mice, the mean RNA levels were 1.57 × 10^4^ ± 0.93 × 10^4^ copies/ml and 1.79 × 10^4^ ± 1.22 × 10^4^ copies/ml plasma for CD4^+^ mice and ZSG mice, respectively. At 24 dpi, the HIV-1 RNA levels in plasma samples from HIV-1-infected ZSG and CD4^+^ mice were 1.14 × 10^5^ ± 1.28 × 10^5^ copies/ml and 1.28 × 10^5^ ± 0.91 × 10^5^ copies/ml plasma, respectively. At 31 dpi, the levels of HIV-1 RNA in plasma samples from HIV-1-infected ZSG and CD4^+^ mice were 2.31 × 10^5^ ± 2.73 × 10^5^ copies/ml and 1.56 × 10^5^ ± 1.37 × 10^5^ copies/ml, respectively. There was no HIV-1 RNA detected in the HIV-1-infected NB-ZSG mice at any time point, and as expected, no HIV-1 RNA was detected in plasma samples from all uninfected mice (Fig. S2). The results indicate that NB-ZSG expression in CD4^+^ cells prior to HIV-1 infection resulted in undetectable HIV-1 RNA in animal plasma for up to 31 dpi.

10.1128/mBio.01769-19.3 FIG S2 No HIV-1 RNA detected in plasma samples from uninfected mice. Analysis of HIV-1 RNA levels in plasma samples from uninfected CD4^+^ cell-engrafted or unmodified mice was performed. CD4^+^_C mice indicates animals engrafted with uninfected CD4^+^ cells. ZSG_C mice indicates animals engrafted with uninfected CD4^+^ cells expressing ZSG. NB-ZSG_C mice indicates animals engrafted with CD4^+^ cells expressing NB-ZSG. blank mice indicates animals engrafted with uninfected and unmodified CD4^+^ cells (*n* = 4 mice for each group). Download FIG S2, EPS file, 0.4 MB. Copyright © 2019 Jin et al. 2019 Jin et al. This content is distributed under the terms of the Creative Commons Attribution 4.0 International license.

### Low levels of HIV-1 mRNA were measured in cells from organs of HIV-1-infected NB-ZSG mice.

**FIG 3:**
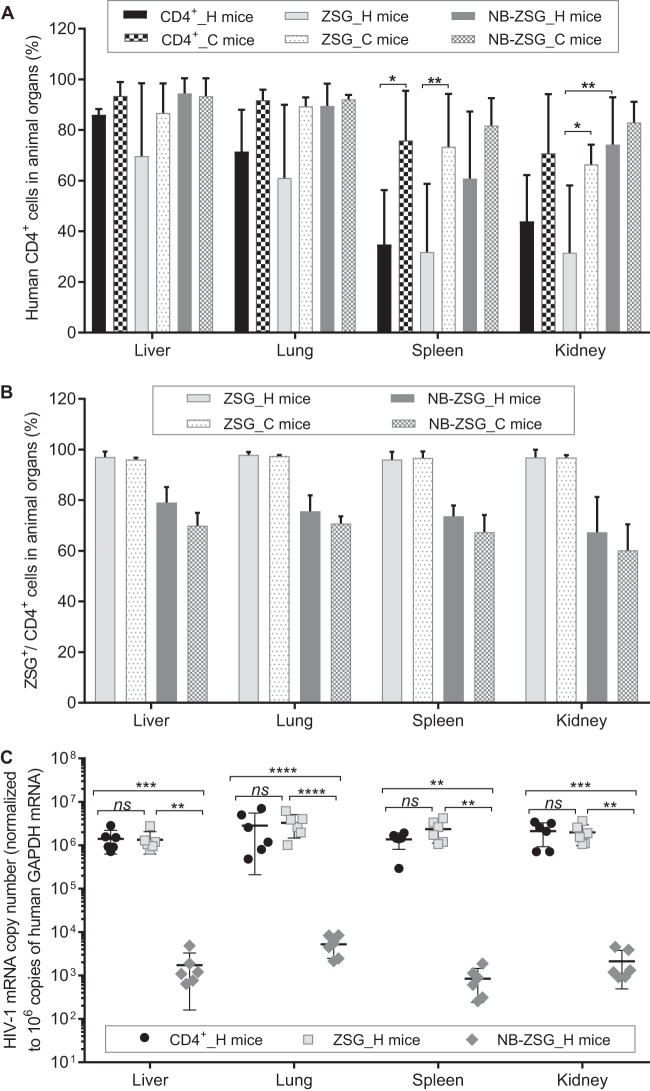
Analysis of organ samples from mice engrafted with CD4^+^ cells expressing NB-ZSG and ZSG and unmodified CD4^+^ cells. Organ cells were collected through a cell strainer and stained with an anti-human CD4-APC antibody. (A and B) Percentage of human CD4^+^ cells (A) and ZSG fluorescence of NB-ZSG and ZSG cells (B) in animal organs were analyzed by flow cytometry. (C) HIV-1 RNA levels were analyzed by RT-qPCR and normalized to human GAPDH mRNA levels in the sample. The key follows the scheme in Fig. 2. The mean values and SD are shown. Statistical significance is indicated (*, *P* < 0.05; **, *P* < 0.01; ***, *P* < 0.001; ****, *P* < 0.0001; *ns*, not statistically different).

All animals were sacrificed at 31 dpi, and samples of the liver, lung, spleen, and kidney were collected. Total cells from organ samples were strained from the tissue and then stained with an anti-human CD4-APC antibody to identify human CD4^+^ cells (Fig. 3A). No significant difference in the percentages of human CD4^+^ cells in liver and lung in all the animal groups was observed, although a trend toward fewer human CD4^+^ cells in these tissues was observed in the HIV-1-infected CD4^+^ mice (designated CD4^+^_H) (Fig. 3A) and ZSG mice (designated ZSG_H) (Fig. 3A). In spleen samples, the levels of CD4^+^ cells in ZSG_H and CD4^+^_H mice were significantly lower than those in uninfected ZSG mice (designated ZSG_C) and CD4^+^ mice (designated CD4^+^_C), whereas there was no significant difference between the NB-ZSG_H and NB-ZSG_C mouse groups. However, there were no significant differences between the NB-ZSG_H mouse group and the ZSG_H and CD4^+^_H mouse groups. In kidney, NB-ZSG_H mice had a significantly higher CD4^+^ cell level than that in ZSG_H mice. The ZSG_H mice had a significantly lower level of CD4^+^ cells than that in ZSG_C mice. There was no significant difference between the NB-ZSG_H and NB-ZSG_C, ZSG_C, and CD4^+^_C mouse groups. The kidneys of NB-ZSG_H mice showed a trend toward higher levels of human CD4^+^ cells than in CD4^+^_H mice, but the difference was not statistically significant. As a whole, the data show that levels of CD4^+^ cells were similar in NB-ZSG mice, irrespective of HIV-1 infection, suggesting that Nullbasic strongly protected CD4^+^ cell viability, which was evident at 31 dpi.

Flow cytometry was used to monitor the expression of ZSG and NB-ZSG by fluorescence. As shown in Fig. 3B, the mean percentage of ZSG-positive cells in organs was >96.0% for all ZSG mice irrespective of HIV-1 infection. For NB-ZSG mice, the mean percentage of NB-ZSG cells ranged from 58% to 78% in the uninfected and infected NB-ZSG mice, which was not statistically different from the levels observed in blood (Fig. 2).

The HIV-1 mRNA levels in the organ samples were analyzed as previously described (18) and normalized to human GAPDH mRNA levels in the same sample. The results showed that NB-ZSG mice had greatly reduced levels of HIV-1 mRNA in all organ samples. As shown in Fig. 3C, the mean HIV-1 mRNA levels in the organs of HIV-1-infected NB-ZSG mice were lower by ∼600 to ∼2,800-fold than those in HIV-1-infected ZSG mice and CD4^+^ mice. The level of inhibition by NB-ZSG observed here was much higher than the level of inhibition observed *in vitro* (Fig. 1A). Finally, and as expected, no human GAPDH mRNA signal was detected in mouse organ samples from nonengrafted mice (Fig. S3A), and HIV-1 mRNA was not detected in any uninfected mouse (Fig. S3B).

10.1128/mBio.01769-19.4 FIG S3 Specific detection of human GAPDH mRNA and lack of HIV-1 mRNA in RNA samples taken from uninfected mice. (A) Analysis of human GAPDH mRNA in RNA samples from CD4^+^ cell-engrafted or unmodified mouse liver, lung, spleen, and kidney by RT-qPCR. (B) Analysis of HIV-1 mRNA levels in RNA samples from uninfected CD4^+^ cell-engrafted or unmodified mice. CD4^+^_C mice indicates animals engrafted with uninfected CD4^+^ cells. ZSG_C mice indicates animals engrafted with uninfected CD4^+^ cells expressing ZSG. NB-ZSG_C mice indicates animals engrafted with CD4^+^ cells expressing NB-ZSG. blank mice indicates animals engrafted with uninfected and unmodified CD4^+^ cells (*n* = 4 mice for each group). Download FIG S3, EPS file, 1.1 MB. Copyright © 2019 Jin et al. 2019 Jin et al. This content is distributed under the terms of the Creative Commons Attribution 4.0 International license.

In summary, NSG mice engrafted with human CD4^+^ cells transduced with NB-ZSG prior to HIV-1 infection had undetectable viral RNA in plasma, whereas high levels of viral RNA were detected in the plasma samples of control mice. In NB-ZSG mice, the levels of HIV-1 mRNA in CD4^+^ cells isolated from the organs showed significant reductions compared to control animals.

### Postinfection treatment of CD4^+^ cells with NB-ZSG delays HIV-1 replication.

**FIG 4:**
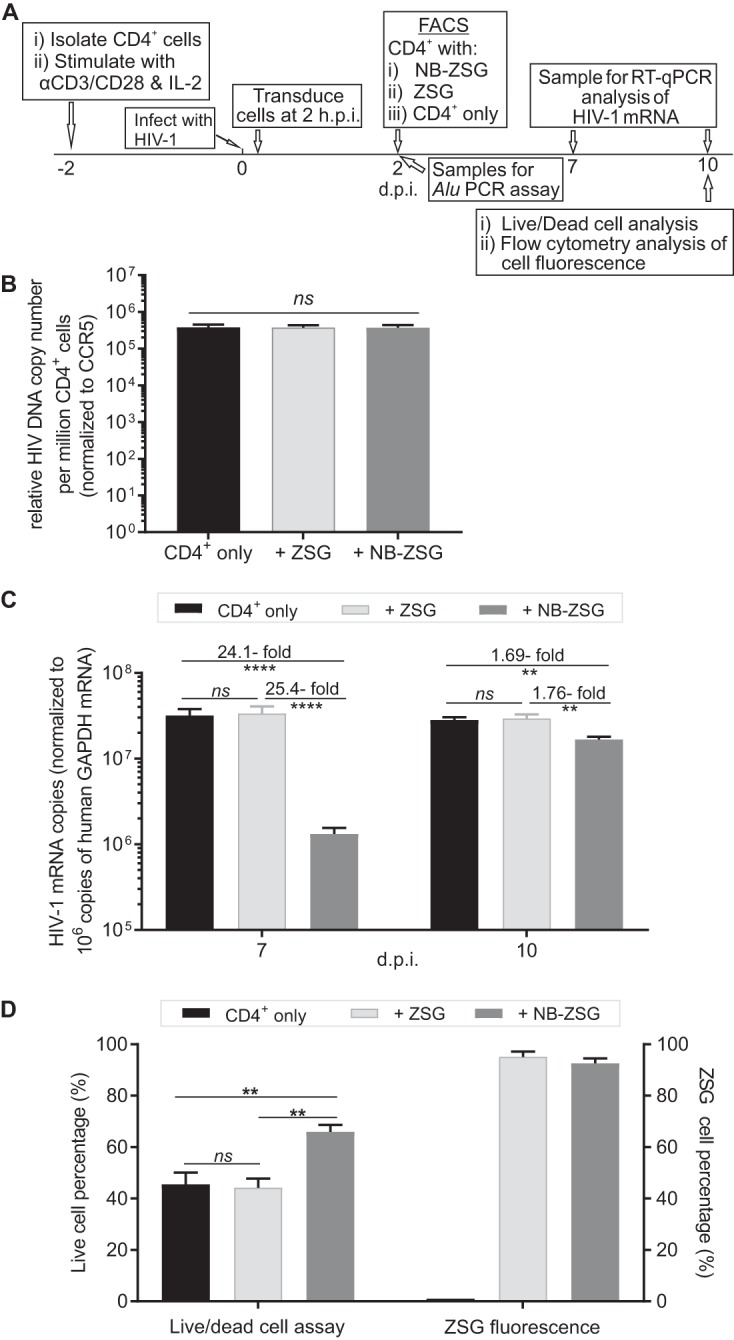
Analysis of postinfection-treated CD4^+^ cells expressing NB-ZSG or ZSG and unmodified HIV-1-infected CD4^+^ cells *in vitro*. (A) Schematic of the postinfection treatment experiment. Briefly, activated CD4^+^ cells were infected with HIV-1 for 2 h. The cells were transduced using VLPs conveying genes encoding NB-ZSG or ZSG. (B) ZSG- and NB-ZSG-positive cells were collected by FACS selection at 2 dpi, and a cell sample was collected for analysis of integrated HIV-1 DNA. (C) The rest of the cells were expanded in medium containing IL-2, and the bulk of the cells were used to engraft mice at 4 dpi. A sample of the sorted cells was grown *in vitro* for up to 10 dpi to measure HIV-1 mRNA, live/dead cell percentages, and fluorescence of ZSG and NB-ZSG in cells. HIV-1 mRNA levels were analyzed by RT-qPCR and normalized to human GAPDH mRNA levels in the sample. (D) HIV-1-infected CD4^+^ cells were analyzed with a live/dead cell assay at 10 dpi. The fluorescence of NB-ZSG- and ZSG-transduced cells was monitored by flow cytometry. Samples were assayed in duplicate from three independent assays. The mean values and SD are shown. Statistical significance is indicated (**, *P* < 0.01; ****, *P* < 0.0001; *ns*, not statistically different). h.p.i., hours postinfection.

We then investigated if Nullbasic could inhibit virus replication in human primary CD4^+^ cells that were infected by HIV-1 first. The experimental scheme is shown in Fig. 4A. Activated CD4^+^ cells were infected with HIV-1 for 2 h and then transduced with NB-ZSG or ZSG virus-like particles (VLPs). After 48 h, the NB-ZSG- and ZSG-positive cells were enriched by FACS selection, and cells were collected to measure integrated HIV-1 DNA levels in the cells with an *Alu* PCR assay. There was no significant difference in the levels of integrated HIV-1 DNA among the three cell groups (Fig. 4B). Overall, the level of integrated DNA measured in this postinfection treatment model was ∼500-fold higher than that in the preinfection treatment model. We measured NB-ZSG and ZSG mRNA levels in the different cell groups at 7 dpi using oligonucleotide primers specific for either the NB-ZSG or ZSG sequence as described above. As shown in Fig. S4, the relative levels of NB-ZSG mRNA measured were similar irrespective of the primers used in the RT-qPCR assay. Also, the levels of NB-ZSG mRNA measured here were similar to those in cells in the preinfection treatment scenario (Fig. S1A).

10.1128/mBio.01769-19.5 FIG S4 NB-ZSG mRNA levels in HIV-1-infected CD4^+^ cells. Cellular RNA was extracted from HIV-1-infected cells at 7 dpi. The level of NB-ZSG mRNA in the sample was measured by RT-qPCR. Oligonucleotide primers specific for NB and ZSG sequences were used separately. NB-ZSG mRNA levels were normalized to human GAPDH mRNA levels in the samples. CD4^+^ only indicates HIV-1-infected and unmodified CD4^+^ cells. + ZSG indicates HIV-1-infected CD4^+^ cells treated with ZSG VLPs. + NB-ZSG indicates HIV-1-infected CD4^+^ cells treated with NB-ZSG VLPs. The results are presented as mean values ± SD. Download FIG S4, EPS file, 0.3 MB. Copyright © 2019 Jin et al. 2019 Jin et al. This content is distributed under the terms of the Creative Commons Attribution 4.0 International license.

Animals were engrafted with the cells at 4 dpi (2 days after FACS analysis). An aliquot of cells was grown *in vitro* and assayed at 7 and 10 dpi for viral mRNA. As shown in Fig. 4C, the level of HIV-1 mRNA at 7 dpi, normalized to the GAPDH mRNA level in the same sample, in NB-ZSG-treated cells was reduced by ∼24- to 25-fold compared to those in ZSG-treated and untreated CD4^+^ cells and was reduced by <2-fold at 10 dpi.

Cell samples collected at 10 dpi were also assayed for the percentages of live cells and ZSG fluorescence-positive cells. At 10 dpi, the live-cell percentage of control cells, at ∼45%, was significantly lower than that of NB-ZSG-treated cells, at ∼66% (Fig. 4D), which was similar to the live-cell percentage observed in uninfected CD4^+^ cells (Fig. 1D). The percentages of ZSG-positive cells were ∼92% for NB-ZSG-treated cells and ∼95% for ZSG-treated cells. The data indicate that NB-ZSG delayed HIV-1 replication and improved levels of live CD4^+^ cells at 10 dpi compared to control cells.

### NB-ZSG delays HIV-1 replication in NSG mice in the postinfection treatment model.

**FIG 5:**
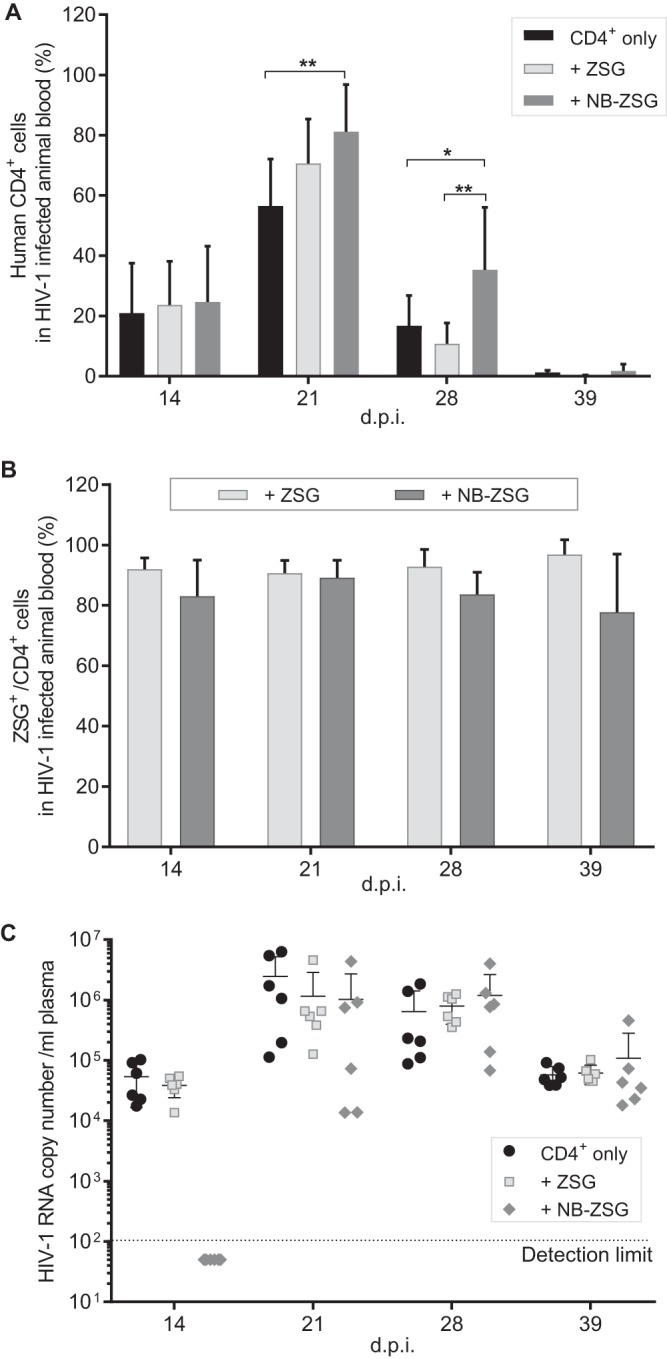
Analysis of postinfection treatment blood samples from mice engrafted with CD4^+^ cells. Animals were engrafted with 4 million HIV-1-infected human CD4^+^ cells, which were unmodified CD4^+^ cells or CD4^+^ cells that expressed ZSG or NB-ZSG. (A) Blood samples were taken weekly from each animal and analyzed for the percentage of human CD4^+^ cells in samples using an anti-human CD4-APC antibody. (B) The same cell samples were analyzed for fluorescence of ZSG or NB-ZSG. (C) HIV-1 RNA levels in plasma samples were quantified by RT-qPCR for up to 39 dpi. The mean values and SD are shown. Statistical significance is indicated (*, *P* < 0.05; **, *P* < 0.01; *ns*, not statistically different).

Two days after the FACS analysis, 4 million NB-ZSG- and ZSG-treated or untreated cells were injected into groups of 6 animals for engraftment. Blood samples were taken at 14, 21, 28, and 39 dpi. As shown in Fig. 5A, human CD4^+^ cells were detected at 14 dpi in blood samples, which reached a peak level at 21 dpi and decreased thereafter. However, in NB-ZSG-treated mice, the CD4^+^ cell percentage in blood samples was significantly higher than that in untreated CD4^+^ mice at 21 dpi and was significantly higher than those in both ZSG-treated mice and untreated mice at 28 dpi. At 39 dpi, the percentage of human CD4^+^ cells in blood was below 6.8% in all animals, and no significant difference in the percentages of CD4^+^ cells in the blood was observed.

The ZSG fluorescence in NB-ZSG and ZSG CD4^+^ cells was analyzed by cell flow cytometry. The mean percentages of ZSG fluorescence-positive cells in NB-ZSG-treated animals were above 80% at 14, 21, and 28 dpi and decreased to ∼78% at 39 dpi. The percentages of ZSG fluorescence-positive cells in ZSG-treated animals were above 90% at all time points, as shown in Fig. 5B. Overall, a trend toward lower levels of NB-ZSG cells than of ZSG cells was noted in the animal groups, but this difference was not statistically significant.

The HIV-1 RNA levels in the plasma samples were measured by RT-qPCR (Fig. 5C). At 14 dpi, no HIV-1 RNA was detected in the blood samples from NB-ZSG-treated CD4^+^ cell-engrafted animals, whereas means of ∼3.9 × 10^4^ copies/ml HIV-1 RNA were detected in ZSG-treated CD4^+^ cell-engrafted animals and ∼5.4 × 10^4^ copies/ml HIV-1 RNA were detected in the nontreated CD4^+^ cell-engrafted animals. However, at 21 dpi, HIV-1 RNA was detected in all animal groups. The mean levels were ∼2.5 × 10^6^ copies/ml, *∼*1.2 × 10^6^ copies/ml, and ∼1.0 × 10^6^ copies/ml HIV-1 RNA in blood samples from nontreated CD4^+^ cell-engrafted and ZSG-treated and NB-ZSG-treated CD4^+^ cell-engrafted animals, respectively. At 28 dpi, the HIV-1 RNA levels decreased slightly in the untreated and ZSG mice, whereas plasma samples from NB-ZSG mice had a mean of ∼1.2 × 10^6^ copies/ml. At 39 dpi, the HIV-1 RNA levels decreased in all animal groups by 10- to 40-fold from peak levels.

In summary, NB-ZSG mice demonstrated a delay in HIV-1 replication and increased levels of circulating CD4^+^ cells at 21 dpi compared to CD4^+^-only mice (nontreated CD4^+^ cell-engrafted mice) and at 28 dpi compared to both ZSG and CD4^+^-only mice. There was no significant difference in viral RNA levels between animal groups after 14 dpi.

### Trend toward reduced viral mRNA by NB-ZSG in human CD4^+^ cells in organs.

The animals engrafted with untreated CD4^+^ or NB-ZSG- or ZSG-treated CD4^+^ cells were sacrificed at 39 dpi. Organs were harvested to measure the percentage of human CD4^+^ cells residing in tissue and the level of HIV-1 mRNA in the samples.

**FIG 6:**
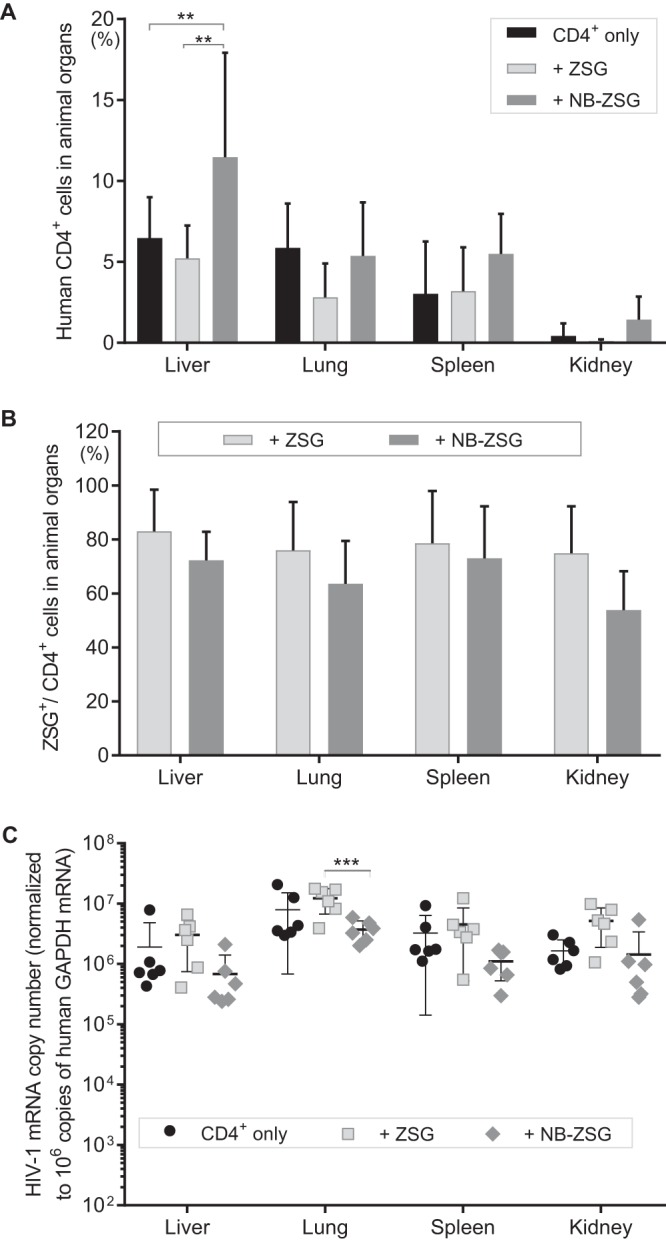
Analysis of postinfection treatment organ samples from mice engrafted with CD4^+^ cells expressing NB-ZSG and ZSG and with unmodified HIV-1-infected CD4^+^ cells. Organ cells were collected through a cell strainer and stained with an anti-human CD4-APC antibody. (A and B) The percentage of human CD4^+^ cells (A) and the fluorescence of NB-ZSG- or ZSG-transduced CD4^+^ cells (B) in animal organs were analyzed by flow cytometry at 39 dpi. (C) HIV-1 mRNA levels were analyzed by RT-qPCR and normalized to human GAPDH mRNA levels in the same sample. The mean values and SD are shown. Statistical significance is indicated (**, *P* < 0.01; ***, *P* < 0.001).

As shown in Fig. 6A, the level of human CD4^+^ cells in all organ samples was below 20%. The mean CD4^+^ cell level in liver of the NB-ZSG mice, at ∼11%, was significantly higher than those in ZSG mice, at ∼5%, and CD4-only mice, at ∼6%. However, no significant differences in CD4^+^ cell levels were observed in lung, spleen, and kidney samples for the animal groups. The mean levels of ZSG-fluorescent CD4^+^ cells (Fig. 6B) in the organ samples from NB-ZSG mice were ∼72% in the liver, ∼64% in the lung, ∼73% in the spleen, and ∼53% in the kidney, and slightly higher mean levels of ZSG-fluorescent CD4^+^ cells were observed in organ samples from ZSG mice, at ∼83% in liver, ∼76% in lung, ∼79% in spleen, and ∼75% in the kidney.

HIV-1 mRNA levels in the cells from organ samples were measured by RT-qPCR and normalized to human GAPDH mRNA levels in the same sample. The relative mean levels of HIV-1 mRNA in cells from liver, lung, spleen, and kidney of NB-ZSG mice trended toward reduced levels compared to those in samples from ZSG mice and CD4^+^-only mice. Of these, only the lung samples from NB-ZSG mice had a significant reduction of HIV-1 mRNA compared to the samples from ZSG mice and CD4^+^ mice. However, no other significant differences in viral mRNA levels among liver, spleen, and kidney were observed. Liver samples from NB-ZSG and ZSG HIV-1-infected mice were also analyzed by RT-qPCR, as described above, for the expression of NB-ZSG and ZSG, respectively. The mean level of NB-ZSG mRNA was ∼1.5 × 10^5^ copies (per 10^6^ copies of GAPDH mRNA) (Fig. S5), which is comparable to levels of NB-ZSG mRNA measured in CD4^+^ cells at 7 dpi (Fig. S4). The mean level of ZSG mRNA in CD4^+^ cells was ∼2- to 3-fold higher than that of NB-ZSG, which was also observed in the preinfection treatment model (Fig. S1). No ZSG or NB-ZSG mRNA was detected in samples from HIV-1-infected CD4^+^-only mice (Fig. S5).

10.1128/mBio.01769-19.6 FIG S5 NB-ZSG mRNA levels in HIV-1-infected CD4^+^ cells from mouse liver. Cellular RNA was extracted from liver samples of HIV-1-infected animals at 39 dpi. The level of NB-ZSG mRNA in the sample was measured by RT-qPCR. Oligonucleotide primers specific for NB and ZSG sequences were used separately. NB-ZSG mRNA levels were normalized to human GAPDH mRNA levels in the samples. CD4^+^ only_mice indicates animals engrafted with HIV-1-infected and unmodified CD4^+^ cells. + ZSG_mice indicates animals engrafted with HIV-1-infected CD4^+^ cells treated with ZSG VLPs. + NB-ZSG_mice indicates animals engrafted with HIV-1-infected CD4^+^ cells treated with NB-ZSG VLPs. The results are presented as mean values ± SD. Download FIG S5, EPS file, 0.3 MB. Copyright © 2019 Jin et al. 2019 Jin et al. This content is distributed under the terms of the Creative Commons Attribution 4.0 International license.

Overall, the combined data show that in the postinfection treatment model, NB-ZSG delayed virus replication and improved CD4^+^ cell levels in blood and liver of HIV-1-infected mice, but it did not significantly reduce HIV-1 mRNA levels in cells or virus levels in the blood after 14 dpi.

## DISCUSSION

The Tat mutant variant called Nullbasic is a unique anti-HIV-1 agent because it can inhibit three different steps of viral replication: synthesis of viral mRNA by RNA polymerase II, reverse transcription of viral RNA into DNA, and Rev-mediated transport of viral mRNA (16). Our previous studies also showed that NB-ZSG inhibited HIV-1 replication in primary human CD4^+^ cells (21, 22), with no effects on CD4^+^ cell viability with respect to levels of apoptosis, cell metabolic activity by a 3-(4,5-dimethylthiazol-2-yl)-5-(3-carboxymethoxyphenyl)-2-(4-sulfophenyl)-2H-tetrazolium (MTS) assay, or cell proliferation *in vitro* (21). Given these positive results, we further investigated Nullbasic as a possible gene therapeutic agent *in vivo* using NSG mice engrafted with human CD4^+^ cells.

Here, retroviral vector-mediated gene transfer of Nullbasic in human CD4^+^ cells engrafted in a mouse model demonstrated strong inhibition of virus replication *in vivo* in a preinfection treatment model. In a challenging postinfection treatment model, Nullbasic delayed virus replication and improved CD4^+^ cell levels. There are several differences in the two models used in this study that may explain the observed differences in the inhibition of HIV-1 replication. The preinfection treatment model represents a typical gene transfer scenario where uninfected cells are treated via transduction and then challenged by virus. In this case, the inhibitor was delivered prior to infection and had time to express sufficient levels of the antiviral protein required for effective virus control (16, 17, 19, 20, 22). This is important with respect to Nullbasic because it inhibits HIV-1 replication in a dose-dependent manner (16, 18). For example, the inhibitory effect of Nullbasic on HIV-1 can be reversed by siRNAs that downregulate NB-ZSG expression in HIV-1-infected Jurkat cells (18). Our experience is that robust expression of NB-ZSG posttransduction requires at least 2 days. This is evident in CD4^+^ cells that expressed high levels of NB-ZSG prior to HIV-1 infection and were highly resistant to HIV-1 replication, as indicated by strongly reduced levels of HIV-1 mRNA in the cells. However, the postinfection treatment model allowed for HIV-1 replication in an activated CD4^+^ T cell population for 48 h prior to FACS selection of NB-ZSG-positive CD4^+^ cells, where the transduction rate of retroviral vector gene transfer was ∼30 to 40% in the HIV-1-infected CD4^+^ cells. Here, a number of cells would support productive virus replication. Also, Tat is made by infected cells during this time, which can be secreted by HIV-1-infected cells (26–28). It has been estimated that about two-thirds of all cellular Tat produced may be secreted by primary human CD4^+^ T cells *in vitro* (29), which can affect other cells (28, 30, 31). The *Alu* PCR results (Fig. 4B) also confirmed that the level of integrated HIV-1 DNA in the postinfection treatment cells was 3 logs higher than that in the preinfection treatment cells (Fig. 1B). Nevertheless, in this challenging postinfection treatment model, NB-ZSG reduced virus mRNA levels in cells by ∼25-fold *in vitro* at 7 dpi, delayed a detectable viral load by 14 days *in vivo*, and imparted a trend toward improved CD4^+^ cell levels and lower viral mRNA levels both *in vitro* and *in vivo*, although *in vivo*, this was statistically significant only in the liver for increased CD4^+^ cell numbers and in the lung for reduced viral mRNA. We noted higher levels of human cell engraftment in preinfection-treated cells than in postinfection-treated cells, which was evident in both blood and tissue samples. Several factors could have affected the levels of human cell engraftment in mice. For example, *in vitro*, the postinfection-treated CD4^+^ cell population had 12- to 15-fold higher levels of HIV-1 mRNA at 7 dpi and increased levels of dead cells at 10 dpi compared to the preinfection-treated CD4^+^ cell population. Animals that were engrafted with these postinfection treated cell populations showed increased viral loads in plasma samples compared to preinfection-treated CD4^+^ cells, especially before 28 dpi. In addition, subtle differences in the way in which cells were handled, such as when the cells underwent FACS analysis, could have an effect on human cell engraftment levels in mice.

In this study, we did not observe that NB-ZSG had a significant adverse effect on human CD4^+^ cell engraftment of NSG mice, as human CD4^+^ cell levels were not significantly different among the uninfected animal groups. In both models, CD4^+^ cells were detectable in the blood sample between 7 and 10 days and reached a peak between 14 and 17 days after the engraftment. The levels of human CD4^+^ cell populations in NB-ZSG mice were generally the same as or higher than those in infected control animals. This is in line with our previous results of cytotoxic assays *in vitro* where no obvious negative effect of Nullbasic on host cells was observed (21, 22).

The retention and expression of the transferred gene were confirmed by the detection of ZSG fluorescence and NB-ZSG mRNA in CD4^+^ cells. In both models, the ZSG-positive cell levels were generally over 90% for the ZSG vector throughout the experiment period but varied between 48.5% and 98.5% for the NB-ZSG vector in blood cells during the experiment. The level of NB-ZSG-positive cells decreased from >90% to between 55 and 80% in CD4^+^ cells detected in organ tissue. We previously observed similar decreasing expression of NB-ZSG in CD4^+^ cells *in vitro* over time (21, 22). The percentage of ZSG-fluorescent cells in NB-ZSG mice decreased over time compared to ZSG mice. Nevertheless, in the preinfection treatment model, stable expression of NB-ZSG was observed in >50% of the engrafted NB-ZSG CD4^+^ cells for up to 31 dpi. Why the percentage of NB-ZSG-positive CD4^+^ cells trended lower over time is unknown, but we suspect that stable expression of NB-ZSG is regulated by a combination of factors that may include the nature of the retroviral integration site in cellular DNA, the activation state of the cell, and effects of NB-ZSG on cellular factors. It is intriguing that in the postinfection treatment model, where viral loads were high, NB-ZSG expression was maintained in ∼80% of the CD4^+^ cells over 39 dpi.

Tat is essential for HIV-1 replication because it stimulates transcription from the viral promoter by interaction with P-TEFb and then binding to the trans-activation response element (TAR) RNA stem-loop structure in the nascent RNA transcript (32). The role of Tat in HIV-1 transcription can be inhibited directly by didehydro-cortistatin A (dCA), which binds to the basic domain in Tat and inhibits its interaction with HIV-1 TAR RNA (33), whereas Nullbasic is believed to inhibit HIV-1 transcription by binding to P-TEFb (19, 34). *In vitro*, dCA was reported to inhibit HIV-1 replication in acutely infected human peripheral blood mononuclear cells (PBMCs), with a maximum inhibition plateau of 86%, and it inhibited virus production by CD4^+^ T cells isolated from HIV-1-infected patients by ∼25% (35). However, dCA is a very strong inhibitor of HIV-1 reactivation from latency both *in vitro* (36) and *in vivo* (37). Interestingly, viral rebound of HIV-1 in latently infected cells is suppressed even after dCA treatment is discontinued, suggesting that dCA causes prolonged transcriptional silencing of the viral promoter. While Nullbasic has demonstrated strong inhibition of HIV-1 reactivation in cell line models of viral latency, further work is required to determine if Nullbasic has a similar capacity to inhibit HIV-1 reactivation in primary cell models of HIV-1 latency *in vitro* and *in vivo*. We are currently testing the delivery of Nullbasic protein to human PBMCs using nanoparticle technologies, which should help to address these questions.

The gene therapeutic approach to cure HIV-1 remains an important area of research because of the potential for long-term, durable antiviral effects by a single treatment. Given the remarkable inhibition observed in this simple animal model of an acute and active HIV-1 infection, as a gene therapy candidate, Nullbasic could be a part of a functional-cure strategy.

## MATERIALS AND METHODS

### Mice.

NOD.Cg-*PrkdcscidIL2rgtmlWjl*/Sz (NOD/SCID/IL2Rγ^null^ [NSG]) mice were bred in-house (QIMR Berghofer Medical Research Institute, Brisbane, QLD, Australia). Mice were housed in sterile microisolator cages and received autoclaved water supplemented with 2 ml/liter Baytril 50 water (Bayer Australia Ltd., Pymble, NSW, Australia) and normal chow. All mouse work was conducted in accordance with the “Australian code for the care and use of animals for scientific purposes” as defined by the National Health and Medical Research Council of Australia. All animal procedures were performed in accordance with protocols approved by the QIMR Berghofer Medical Research Institute Animal Ethics Committee (A1306-602M), and HIV-1 work was conducted in a biosafety level 3 facility.

### Cell lines and PBMCs.

HEK293T (ATCC) and Phoenix-Ampho (38) cell lines were grown in Dulbecco’s modified Eagle’s medium (DMEM; Life Technologies, Carlsbad, CA) supplemented with 10% (vol/vol) fetal bovine serum (FBS), penicillin (100 IU/ml), and streptomycin (100 μg/ml) (referred to as DF10 medium).

Peripheral blood mononuclear cells (PBMCs) were isolated from a healthy donor’s buffy coat supplied by the Australian Red Cross Blood Service using Ficoll density gradient centrifugation as previously described (21, 22). Briefly, CD4^+^ cells were isolated from the PBMCs by using an EasySep human CD4^+^ T cell isolation kit (Stemcell Technologies Australia Pty. Ltd., Tullamarine, VIC, Australia) according to the manufacturer’s instructions. The selected cells were stimulated in a precoated 3-cm tissue culture dish with purified anti-human CD3 (clone HIT3a) and anti-human CD28 (clone CD28.2) antibodies (BioLegend, San Diego, CA) in RPMI medium supplemented with 20% (vol/vol) FBS and 5 ng/ml interleukin-2 (IL-2) (referred to as RF20 IL-2 medium) for 2 days. All cells were maintained at 37°C in humidified incubators with 5% CO_2_.

### HIV-1 and virus-like particle production.

HIV-1_NL4-3_ stocks were made by transfection of HEK293T cells with a proviral DNA plasmid (39, 40). The medium was replaced at 24 h posttransfection, and HIV-1 was harvested at 72 h posttransfection. All virus stocks were quantified by a CAp24 enzyme-linked immunosorbent assay (ELISA) and stored in small aliquots at −80°C as previously described (18).

VLPs that convey NB-ZSG or ZSG were produced as previously described (21). Briefly, 10 μg of the pSRS11-SF-γC vector (25) expressing NB-ZSG or ZSG and 2 μg of a plasmid expressing Gag-Pol were transfected into Phoenix-Ampho cells (41) with X-tremeGENE HP DNA transfection reagent (Roche Diagnostics GmbH, Mannheim, Germany) in accordance with the manufacturer’s protocol. The cell medium was replaced at 24 h posttransfection. VLPs were harvested by filtration with a sterile 0.22-μm syringe filter (Sartorius Stedim Biotech GmbH, Göttingen, Germany) at 48 h and 72 h posttransfection.

### CAp24 ELISA.

An HIV-1 CAp24 ELISA (ZeptoMetrix Corp., Buffalo, NY) was used to measure CA levels in culture supernatants in accordance with the manufacturer’s instructions.

### Transduction of CD4^+^ T cells.

NB-ZSG or ZSG VLP supernatants (10 ml) were concentrated by using a precipitation method with 8.5% (vol/vol) polyethylene glycol 6000 (Sigma-Aldrich, St. Louis, MO) and 0.3 M NaCl as described previously (42). The mixture was incubated at 4°C for 1.5 h, mixed every 30 min, and then centrifuged at 1,500 × *g* for 1 h at 10°C. The supernatant was discarded, and the precipitate was resuspended in 1 ml RF20 IL-2 medium. The concentrated VLPs (250 μl) were added to a well of a RetroNectin (5 μg/well) (Scientifix Pty. Ltd., South Yarra, VIC, Australia)-coated 24-well plate and incubated at 37°C for 30 min. A total of 5 × 10^5^ stimulated CD4^+^ cells (1 ml) were added to each well and incubated for 2 days. ZSG-positive and -negative cells were sorted and collected by FACS selection with an Aria III cell sorter and rested for 2 days prior to HIV-1 infection.

One million of the sorted CD4^+^ T cells (NB-ZSG or ZSG CD4^+^ cells or CD4^+^ cells only) were infected with HIV-1 at 1 ng CA/million cells/ml for 2 h at 37°C. The cells were then washed with phosphate-buffered saline (PBS) and cultured in RF20 IL-2 medium. Aliquots of 1 × 10^6^ cells were collected from the sorted cells at 16 h p.i. for *Alu* PCR analysis. The cells were then collected at 2 dpi by centrifugation at 300 × *g* for 5 min, washed once with PBS, and resuspended in PBS at 2 × 10^7^ cells/ml for animal injection. Aliquots of the infected cells and uninfected cells were maintained in RF20 IL-2 medium at 37°C. Cell samples were collected at 3, 7, and 10 dpi for monitoring HIV-1 infection, and cell samples collected at 10 dpi were also used for analyzing NB-ZSG expression and cell viability.

### Transduction of HIV-1-infected CD4^+^ T cells.

The activated CD4^+^ cells were infected by HIV-1 at 1 ng CA/million cells/ml for 2 h at 37°C. The infected cells were then washed with PBS and resuspended in RF20 IL-2 medium. The cells were then transduced as described above for the uninfected CD4^+^ cells. Two days after transduction, the ZSG-positive and -negative cells were collected by FACS selection with an Aria III cell sorter. Aliquots of 1 × 10^6^ cells were collected from the sorted cells for *Alu* PCR analysis. The rest of the sorted cells were rested in RF20 IL-2 medium for 2 days. The cells were then collected by centrifugation, washed with PBS, and resuspended in PBS at 2 × 10^7^ cells/ml for animal injection. Aliquots of the infected cells and uninfected cells were maintained in RF20 IL-2 medium at 37°C. Cell samples were collected at 7 and 10 dpi for monitoring HIV-1 infection, and cell samples collected at 10 dpi were also used for analyzing NB-ZSG expression and cell viability.

### Live-cell percentage.

The cell viability of HIV-1-infected CD4^+^ cells collected at 10 dpi was analyzed by using Live/Dead fixable dead cell stain kits (catalog no. L34963; Thermo Fisher Scientific, Waltham, MA) according to the manufacturer’s instructions and by flow cytometry.

### Animal engraftment and sample collection.

Animals were engrafted as previously described, with modifications (23, 24). Briefly, animals were irradiated at 250 cGy and rested overnight in a biosafety level 3 facility. Next, 4 million CD4^+^ cells (HIV-1-infected or uninfected NB-ZSG, ZSG, or CD4^+^-only cells) in 200 μl PBS were injected intravenously. Four mice per group were engrafted with uninfected CD4^+^ cells, and 6 mice per group were engrafted with HIV-1-infected CD4^+^ cells. Four mice were included in the experiment as blank controls. All animals were maintained in a biosafety level 3 animal facility and monitored daily. Blood samples were collected by tail vein bleeding. Approximately 50 μl of blood was collected into an EDTA-coated tube and centrifuged at 500 × *g* for 5 min. The plasma samples were removed to a new tube with the addition of 1 ml TRIzol reagent. The sample was then stored at −80°C for analysis of HIV-1 RNA. The cells were resuspended into 50 μl PBS and stained with anti-human CD4-APC (Miltenyi Biotec, Macquarie Park, NSW, Australia) in the dark at room temperature for 15 min, and the cells were then lysed with 2 ml of BD FACS lysis solution and incubated in the dark for another 15 min. Next, the cells were collected by centrifugation at 500 × *g* for 5 min, washed once with PBS, and resuspended in 100 μl PBS containing 25 mM EDTA and 1% formaldehyde.

At the end of the experiment, animals were sacrificed, and organ tissue (liver, lung, spleen, and kidney) was collected for analysis of human CD4^+^ cell populations and HIV-1 RNA levels in the cells collected from tissue. Cells from the organs were collected by passage through a 70-μm cell strainer and washed once with PBS containing 1% bovine serum albumin (BSA). The cells were then aliquoted for cell staining using anti-human CD4^+^-APC (Miltenyi Biotec, Macquarie Park, NSW, Australia) as described above and for RNA extraction.

### Flow cytometry analysis.

Data from a minimum of 10,000 single cells were collected from each cell sample using a BD LSR 4 flow cytometer. Data were analyzed by FlowJo version 9 single-cell analysis software.

### *Alu* PCR.

*Alu* PCR was performed as described previously, using Fast Start TaqMan master mix (2×; Sigma-Aldrich, St. Louis, MO) (18). Genomic DNA (0.1 μg) was used for a first-round PCR in a 25 μl reaction mixture volume with HIV-1 first-strand forward primer 5′-GGTCTCTCTGGTTAGACCAA and *Alu* reverse primer 5′-TGCTGGGATTACAGGCGTGAG. The reaction mixture underwent 1 cycle of 95°C for 10 min; 25 cycles of 95°C for 30 s, 52°C for 20 s, and 72°C for 2 min; and 1 cycle of 72°C for 7 min. Next, 5 μl of the PCR product was used in a nested-PCR assay. The nested PCR was performed using an HIV-1 forward primer (5′-AACTAGGGAACCCACTGCTTAAG), an HIV-1 reverse primer (5′-TGGTTCTACTTTCGCTTTCGC), and a TaqMan probe (5′–6-carboxyfluorescein–CGGTCGAGTGCTTCAAGTAGTGTGTGCCCGTCCGACCG–6-carboxytetramethylrhodamine). The reaction conditions were 1 cycle of 95°C for 2 min and 45 cycles of 95°C for 15 s and 60°C for 1 min. A TaqMan probe-based assay for CCR5 was used to normalize each sample.

### RT-qPCR assays.

RT-qPCR for HIV-1 RNA was performed as previously described (18). Briefly, RNA from samples was isolated with TRIzol reagent (Thermo Fisher Scientific, Waltham, MA) in accordance with the manufacturer’s protocol. All RNA samples from cells were treated with Turbo DNase I (Thermo Fisher Scientific, Waltham, MA). cDNA was made using 500 ng of total RNA, random hexamer primers, and Superscript III reverse transcriptase (Thermo Fisher Scientific, Waltham, MA) in accordance with the manufacturer’s instructions. HIV-1 RNA was quantified by using primers that targeted the HIV-1 5′-R-U5 region (forward primer 5′-GGTCTCTCTGGTTAGACCA and reverse primer 5′-TGGTTCTACTTTCGCTTTCGC). For cell samples, all HIV-1 copy numbers measured were normalized to the level of human GAPDH mRNA in the same sample, which was measured by PCR with the following primers: forward primer 5′-GCAAATTCCATGGCACCGTC and reverse primer 5′-TCGCCCCACTTGATTTTGG. SYBR green master mix (Scientifix Pty. Ltd., South Yarra, VIC, Australia) was used for qPCR.

### Data analysis.

Data were analyzed by using two-way analysis of variance (ANOVA) with Turkey’s multiple-comparison tests using Prism 7 software (GraphPad Software Inc., CA), unless otherwise described. Statistical significance is indicated in the figures. Data are presented as mean values ± standard deviations (SD).

10.1128/mBio.01769-19.1 TEXT S1 RT-qPCR assays of NB-ZSG expression. Download Text S1, DOCX file, 0.01 MB. Copyright © 2019 Jin et al. 2019 Jin et al. This content is distributed under the terms of the Creative Commons Attribution 4.0 International license.
