# Supplementary material for: Strong In Vivo Inhibition of HIV-1 Replication by Nullbasic, a Tat Mutant
Source: mBio. 2019 Aug 27;10(4):e01769-19. doi: 10.1128/mBio.01769-19 (PMC6712395; doi:10.1128/mBio.01769-19)
Supplement: TEXT S1 [file mBio.01769-19-s0001.docx]

**Supplement method**

**RT-qPCR assays of NB-ZSG expression.** Cellular RNA from samples was isolated with TRIzol reagent (ThermoFisher Scientific, Waltham, MA) in accordance with the manufacturer’s protocol. All RNA samples from cells were treated with Turbo DNase I (ThermoFisher, Waltham, MA). cDNA was made using 500 ng of total RNA, random hexamer primers, and Superscript III reverse transcriptase (ThermoFisher Scientific, Waltham, MA) in accordance with the manufacturer’s instructions. NB-ZSG mRNA was quantified by with primers that targeted NB region (forward 5’-GGAGCCAGTAGATCCTAGACT and reverse 5’-CACCGGCGCCACCACCACCA) and primers that targeted ZSG region (forward 5’-CGGCCTGACCAAGGAGATGA and reverse 5’-TCCACCACGCACAGGTTGAT), respectively. NB-ZSG mRNA l measured were normalized to the level of human GAPDH mRNA in the same sample, which was measured by PCR with the primers: forward 5′-GCAAATTCCATGGCACCGTC and reverse 5′-TCGCCCCACTTGATTTTGG. SYBR green master mix (Scientifix, Cheltenham, Australia) was used for qPCR.
